# Immunodominant IgM and IgG Epitopes Recognized by Antibodies Induced in Enterovirus A71-Associated Hand, Foot and Mouth Disease Patients

**DOI:** 10.1371/journal.pone.0165659

**Published:** 2016-11-02

**Authors:** Kam Leng Aw-Yong, I-Ching Sam, Mia Tuang Koh, Yoke Fun Chan

**Affiliations:** 1 Department of Medical Microbiology, Faculty of Medicine, University of Malaya, 50603 Kuala Lumpur, Malaysia; 2 Department of Paediatrics, Faculty of Medicine, University of Malaya, 50603 Kuala Lumpur, Malaysia; Indian Institute of Science, INDIA

## Abstract

Enterovirus A71 (EV-A71) is one of the main causative agents of hand, foot and mouth disease (HFMD). Unlike other enteroviruses that cause HFMD, EV-A71 is more frequently associated with severe neurological complications and fatality. To date, no effective licensed antivirals are available to combat EV-A71 infection. Little is known about the immunogenicity of viral non-structural proteins in humans. Previous studies have mainly focused on characterization of epitopes of EV-A71 structural proteins by using immunized animal antisera. In this study, we have characterized human antibody responses against the structural and non-structural proteins of EV-A71. Each viral protein was cloned and expressed in either bacterial or mammalian systems, and tested with antisera by western blot. Results revealed that all structural proteins (VP1-4), and non-structural proteins 2A, 3C and 3D were targets of EV-A71 IgM, whereas EV-A71 IgG recognized all the structural and non-structural proteins. Sixty three synthetic peptides predicted to be immunogenic *in silico* were synthesized and used for the characterization of EV-A71 linear B-cell epitopes. In total, we identified 22 IgM and four IgG dominant epitopes. Synthetic peptide PEP27, corresponding to residues 142–156 of VP1, was identified as the EV-A71 IgM-specific immunodominant epitope. PEP23, mapped to VP1 41–55, was recognized as the EV-A71 IgG cross-reactive immunodominant epitope. The structural protein VP1 is the major immunodominant site targeted by anti-EV-A71 IgM and IgG antibodies, but epitopes against non-structural proteins were also detected. These data provide new understanding of the immune response to EV-A71 infection, which benefits the development of diagnostic tools, potential therapeutics and subunit vaccine candidates.

## Introduction

Human enterovirus A71 (EV-A71) belongs to the Enterovirus genus within the family of *Picornaviridae*. EV-A71 is a non-enveloped virus with a single stranded, positive-sense RNA genome of approximately 7.4kb. EV-A71 causes mild hand, foot and mouth disease (HFMD), characterized by fever, vesicular lesions on the palms and soles, and oral ulcers. Unlike other enteroviruses that cause HFMD, EV-A71 is more frequently associated with potentially fatal neurological complications such as encephalitis, acute flaccid paralysis and aseptic meningitis, especially in children below 5 years old [1]. EV-A71 infection was first described in California in 1969 [2]. Since then, several large EV-A71 epidemics have been reported in the Asia Pacific region, including Malaysia [3], Singapore [4], Taiwan [5], Japan [6,7], Brunei [8] and China [9–11], raising serious public health concerns. Currently there are no effective licensed antivirals against EV-A71 [12].

The EV-A71 genome comprises a single open reading frame encoding four structural proteins (VP1-VP4) and seven non-structural proteins (2A-2C and 3A-3D), flanked by 5’ and 3’ untranslated regions (UTRs). These structural proteins form an icosahedral structure, with VP1 to VP3 capsid proteins exposed to the surface and responsible for host receptor binding and antigenicity, whereas VP4 is internal [13].

Children between 6 months and 3 years are at highest risk of acquiring EV-71 infection due to lack of protective immunity [14]. Neutralizing antibodies play an important role in immunoprotection from viral infection. Humoral immune responses are elicited when clinical symptoms appear, and 80% of HFMD patients became positive for neutralizing antibodies against EV-A71 one day after the onset of illness [15]. A major neutralisation epitope of EV-A71, named SP70, was first recognised using synthetic peptides and mapped to the VP1 capsid protein at amino acid position 208–222 [16]. Previous studies have identified two main EV-A71 immunogenic sites capable of eliciting strong neutralizing antibodies in rabbit and mice: the epitopes spanning amino acid positions 208–222 in VP1 [16–21] and positions 136–150 in the VP2 capsid protein [22,23]. In EV-A71, the majority of reported epitopes were identified with immunized rabbit and mice antisera [16,22,24], and only one study on EV-A71 immunogenic epitopes has been conducted using human sera [24]. The only known conformational neutralization epitopes are mapped to the conserved glycine at amino acid VP1-145 [25], amino acid positions 59, 62 or 67 of the VP3 “knob” region [26], and amino acid positions 176–190 of VP3 [27]. All the reported EV-A71 epitopes focused only on EV-A71 structural proteins. Therefore, the immunogenicity of EV-A71 non-structural proteins remains unknown.

In the present study, we aimed to characterize human antibody responses against the structural and non-structural proteins of EV-A71 using serum samples from EV-A71-infected children. A total of 63 synthetic peptides (predicted as potential EV-A71 immunogenic epitopes) covering the structural and non-structural proteins of EV-A71 were also screened to identify EV-A71-specific IgM and IgG immunogenic epitopes.

## Materials and Methods

### Human serum specimens

A total of 43 human serum samples collected from children clinically diagnosed with HFMD during HFMD outbreaks in 2000 and 2012–2013 were used in the present study. All serum samples were confirmed as positive for EV-A71 or non-EV-A71 enteroviruses by virus culture or RT-PCR from throat swabs, vesicle swabs and/or rectal swabs. EV-A71 specific-IgM and -IgG antibodies in sera samples were detected using an EV-A71 IgM-capture enzyme-linked immunosorbent assay (ELISA; Beijing Wantai Biological Pharmacy Enterprise CO., Ltd., China) [28], and western blot and virion-based ELISA analyses, respectively. The neutralization activity of antibodies in sera specimens were tested in triplicate and analyzed by microneutralization assay as previously described [29,30]. A sample was considered positive if the neutralizing titer was ≥1:8. Sera from patient with RT-PCR- or culture-confirmed EV-A71 were then divided into 3 groups. Sera positive for EV-A71-specific IgM and IgG, and with neutralization titers <1:8, were grouped as acute infection with no neutralization. Sera with detectable EV-A71-specific IgM and IgG, and neutralization titers of ≥1:32, were grouped as acute infection with high neutralization. Sera negative for EV-A71-specific IgM but positive for EV-A71-specific IgG, with neutralization titers ≥1:8, were grouped as convalescence. The numbers and characteristics of the serum samples in each group are listed in S1 Table.

The non-HFMD samples used as negative controls consisted of 10 residual serum samples previously tested positive for dengue virus IgM. All non-HFMD samples were confirmed negative for anti-EV-A71 IgM and/or IgG (screened by EV-A71 IgM-capture ELISA and western blot analysis). Sera from 5 healthy adult volunteer donors with EV-A71 neutralization titers ≥1:32 were also used. All sera samples were obtained from the diagnostic virology laboratory, University Malaya Medical Centre in Kuala Lumpur. This study was approved by the Medical Ethics Committee of the University Malaya Medical Centre, Kuala Lumpur, Malaysia (reference number: 932.17) and conducted as per the guidelines of the International Committee on Harmonization of Good Clinical Practice (ICH-GCP) and Declaration of Helsinki. Our institution does not require written informed consent for retrospective studies of HFMD and non-HFMD patients’ serum samples.

### Cell lines and viruses

Human rhabdomyosarcoma (RD, ATCC CCL-136) and human embryonic kidney (HEK293, ATCC CRL-1573) cells were cultured in Dulbecco’s Modified Eagle Medium (DMEM) supplemented with 10% fetal bovine serum (FBS). The EV-A71 virus isolate UH1/PM/1997 (GenBank accession number: AM396587) was propagated in RD cells using maintenance medium supplemented with 2% FBS. Virus stock was prepared by harvesting the infected cells, lysing the cells with one round of freeze-thawing and purification by sucrose cushion ultracentrifugation before storage at -80°C. The virus titers were determined by plaque assay.

### Recombinant EV-A71 plasmids

Codon-optimized cDNA clones encoding the entire EV-A71 proteome (both structural and non-structural) based on consensus sequence alignments of different EV-A71 sequences were synthesized (GenScript, USA). An additional cassette with domain linker, StrepTag II, FLAG Tag and 8X His-Tag functions was designed and synthesized. The synthesized DNAs were subcloned into pEGFP-N1 vector to form pEGFP-N1-cassette-VP1, -VP2, -VP3, -VP4, -3C and -3D expression plasmids. The pEGFP-N1-2B, -2C and -3AB expression plasmids had been previously synthesized by our laboratory. The synthesized plasmid DNA was also subcloned into pET-52b(+) to form a pET-52b(+)-2A expression plasmid. All positive clones were screened by restriction enzyme digestion analysis and confirmed by DNA sequencing.

### EV-A71 protein expression and purification from the mammalian expression system

Enhanced green fluorescent protein (EGFP)-expressing recombinant EV-A71 proteins were expressed in HEK293 cells using Lipofectamine LTX reagent (Invitrogen, USA). Cells were transiently transfected with the pEGFP-cassette-VP1, -VP2, -VP3, -VP4, -3C and -3D and pEGFP-N1-2B, -2C and -3AB plasmids with a ratio of plasmid DNA to Lipofectamine LTX reagent of 1:3. Cells were transfected with 16 μg of plasmid DNA per 4 × 10^6^ cells in Opti-MEM (Invitrogen, USA) for 24 hours at 37°C. This was replaced with fresh maintenance medium supplemented with 10% FBS and cells were incubated for a further 24 hours at 37°C.

Recombinant EV-A71-EGFP proteins were harvested at 48 hours post-infection. Cells were washed with PBS twice and lysed with ice cold lysis buffer (20 mM HEPES (pH 7.5), 280 mM KCl, 1 mM EDTA, 1% Triton X-100) containing protease inhibitors (Sigma Aldrich, Germany). The recombinant EV-A71-EGFP protein lysate was purified using the μMACS GFP isolation kit (Miltenyi Biotec, Germany) according to the manufacturer’s instructions. Briefly, the protein lysates were incubated with μMACS anti-GFP microbeads on ice for 30 minutes, then washed, and the denatured protein was eluted with 70 μl of elution buffer. The purified proteins were stored at -20°C for western blot analysis.

### EV-A71 protein expression and purification from the bacterial expression system

Recombinant EV-A71 proteins were expressed in *Escherichia coli* BL21 (DE3) (New England Biolabs, USA) competent cells transformed with the pET-52b(+)-2A expression plasmid. Protein expression was induced with isopropyl-beta-D-thiogalactopyranoside (Vivantis Technologies, Malaysia) and harvested at 4 hours 30 minutes post-induction. Cells were lysed with denatured lysis buffer (8 M urea, 50 mM NaH_2_PO_4_, 300 mM NaCl, 10 mM imidazole, pH 8.0), followed by sonication, and cell debris was removed by centrifugation. The protein lysates were incubated with Profinity IMAC Ni-charged resins (Bio-Rad, USA) at 4°C for 30 minutes, washed twice with denatured washing buffer (8 M urea, 50 mM NaH_2_PO_4_, 300 mM NaCl, 20 mM imidazole, pH 8.0) and the protein was eluted with 4 ml elution buffer (8 M urea, 50 mM NaH_2_PO_4_, 300 mM NaCl, 300 mM imidazole, pH 8.0). Purified protein was then concentrated using Amicon Ultra centrifugal filters (Merck Millipore, USA) and stored at -20°C for western blot analysis.

### SDS-PAGE and western blot analysis

Proteins from purified EV-A71 virions and protein lysates were mixed with Laemmli buffer and separated by 10% SDS-PAGE. The proteins were transferred onto nitrocellulose membranes, which were blocked with 5% skimmed milk in 0.05% Tween-20 phosphate buffered saline (0.05% PBST) for 1 hour at room temperature. The pooled human serum samples were pre-treated additionally with RIDA RF-Absorbens (R-Biopharm AG, Germany) or DTT (Invitrogen, USA) for IgM- and IgG-specific antibody detection, respectively. The membrane was then incubated with 1:5000 diluted anti-GFP-HRP (Miltenyi Biotec, Germany), 1:300 diluted pooled human serum, 1:100 diluted Light Diagnostics EV-A71 monoclonal antibody 3323 (mAb 3323; Millipore, USA) or 1:1000 diluted EV-A71-specific mAb 979 (Millipore, USA) for 1 hour at room temperature, followed by secondary antibody incubation with the 1:5000 diluted HRP-conjugated polyclonal rabbit anti-human IgM (KPL, USA), 1:3000 diluted Amersham ECL human IgG, HRP-linked whole Ab from sheep (GE Healthcare, USA) or 1:5000 diluted HRP-conjugated goat anti-mouse (Gene Tex, USA) antibody. The immunoblot was developed with Clarity Western ECL Substrate (Bio-Rad, USA) and detected by chemiluminescence. The sizes of the protein bands were determined using the Precision Plus Protein WesternC Standard (Bio-Rad, USA).

### Virion-based ELISA and sera isotyping

Polystyrene 96-well Maxisorp Nunc-immuno plates (Thermo Scientific, Denmark) were coated with 10 μg/ml of purified EV-A71 virions. Wells were blocked with 3% bovine serum albumin (BSA) diluted in 0.05% PBST, and incubated at 37°C for 1 hour. Pooled human sera were pre-treated with RIDA RF-Absorbens to obtain IgM specific antibody. The pooled human sera were then diluted at 1:100 to 1:8000 in 1% BSA-0.05% PBST and incubated for 1 hour at 37°C, followed by 1 hour incubation with the 1:5000 diluted HRP-conjugated polyclonal rabbit anti-human IgM (KPL, USA), 1:5000 diluted HRP-conjugated polyclonal rabbit anti-human IgG (Dakocytomation, Denmark) or 1:500 diluted HRP-conjugated isotype IgG1 (Molecular Probe, USA), IgG2 (Invitrogen, USA), IgG3 (Invitrogen, USA), and IgG4 antibodies (Molecular Probe, USA). TMB microwell peroxidase substrate (KPL, USA) was added and the reactions terminated with 1M phosphoric acid. Absorbance was measured at 450 nm with a reference filter of 630 nm. The optical densities (OD) were plotted as means ± standard deviation (SD).

### Peptide-based ELISA

A total of 63 biotinylated peptides consisting of 15-mer peptides (Pepscan, Netherlands) were generated from consensus alignment of different EV-A71 amino acid sequences. The synthetic biotinylated peptides were either potential EV-A71 antibody epitopes predicted by the Emini surface accessibility scale [31] or published EV-A71 epitope sequences (S2 Table and S1 Fig). Peptides were dissolved in dimethyl sulfoxide (DMSO) to obtain a final concentration of 15 μg/ml. All peptides were screened with the human serum in triplicate and analyzed by indirect ELISA. Briefly, streptavidin high binding capacity-coated plates (Pierce Biotechnology, USA) were blocked with 3% BSA diluted in 0.05% PBST, coated with peptides diluted at 1:1000 in 0.05% PBST and incubated at room temperature for 1 hour. Pooled human sera were pre-treated with RIDA RF-Absorbens to obtain specific IgM antibody. The plates were incubated with 1:500 diluted pooled human sera with 1% BSA-0.05% PBST for 1 hour, followed by incubation with the 1:5000 diluted HRP-conjugated polyclonal rabbit anti-human IgM (KPL, USA) or polyclonal rabbit anti-human IgG (1:5000) antibodies for 1 hour. TMB microwell peroxidase substrate (KPL, USA) was added and the reaction was terminated with 1M phosphoric acid. The OD was measured at 450 nm with a reference filter of 630 nm. The cut-off value was calculated as mean OD of the negative controls. If the mean OD of negative controls was lower than 0.105, the value was considered to be 0.105. Data was presented as signal/cut-off (S/CO) values, with a value <2.1 scored as negative, a value of 2.1–4.9 scored as weakly positive, and a value ≥5 scored as strongly positive [24].

### Three-dimensional structure and sequence analysis

The crystal structure of EV-A71 was retrieved from Protein Data Bank (PDB) (identifier 3VBS) and visualized using the UCSF Chimera software version 1.10.1 [32]. For sequence analysis, sequences from different enteroviruses were downloaded from GenBank and aligned using Geneious R6 (Biomatters Ltd., Auckland, New Zealand).

### Statistical analysis

All graphs were plotted and statistical analyses were performed using GraphPad Prism version 5.0 (GraphPad software, La Jolla, CA). One-way ANOVA with the Kruskal-Wallis test was used to compare optical density readings of serum samples tested for EV-A71-specific IgM and IgG antibodies by ELISA. A *p* value of <0.05 was considered as statistically significant.

## Results

### Antigen recognition by EV-A71-infected patient sera

**Fig 1:**
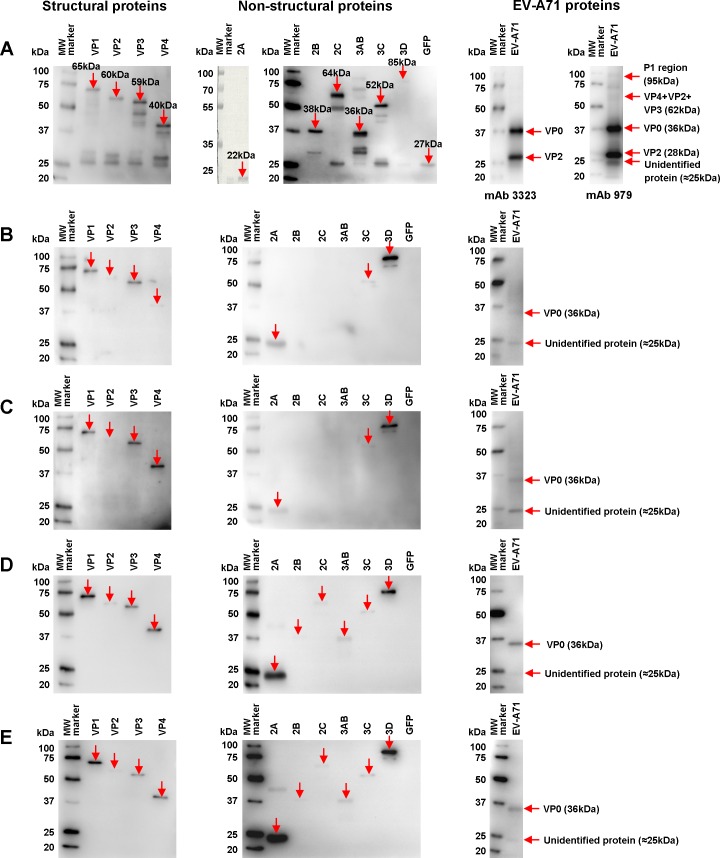
Antigenic profiles of the human anti-EV-A71 antibodies. (A) Control cell lysates were loaded into SDS-PAGE gel electrophoresis. Recombinant EV-A71-EGFP cell lysates (structural and non-structural proteins) were probed with anti-GFP-HRP, while recombinant EV-A71 2A cell lysates were stained with Coomassie brilliant blue R-250. EV-A71 virion proteins were immunodetected with EV-A71-specific mAb 3323 (Millipore, USA) and mAb 979 (Millipore, USA), followed by secondary anti-mouse IgG-HRP. The expected band for each individual recombinant protein is indicated by red solid arrows and the protein sizes are shown. (B) Acute infection with no neutralization sera (n = 2) and (C) acute infection with high neutralization sera (n = 12) were used for EV-A71-specific IgM antibody detection. (D) Acute infection with high neutralization sera (n = 12) and (E) convalescent sera (n = 5) were used for EV-A71-specific IgG antibody detection. An estimated 20 μg of proteins was loaded for SDS-PAGE gel electrophoresis. The amount of EV-A71 structural and non-structural protein cell lysates was normalized with anti-GFP-HRP since the presence of inhibitory factors affected accurate quantitation of total proteins. The EV-A71 protein cell lysates and EV-A71 proteins were subjected to SDS-PAGE gel electrophoresis and probed with pooled human sera at a dilution of 1:300. The immunoblot was developed with Clarity Western ECL substrate and detected by chemiluminescence. Protein bands were determined using the Precision Plus Protein WesternC Standard (Bio-Rad, USA). The antigens recognized by EV-A71-infected patient sera are indicated by red solid arrows.

To define the antigenic recognition profiles of the antibodies from EV-A71-infected patient samples, individual expression plasmids encoding each structural (VP1 to VP4) and non-structural (2A to 3D) gene of the EV-A71 proteins tagged with EGFP were generated. After transient transfection in HEK293 cells, all recombinant EV-A71 EGFP proteins were successfully expressed, as shown by immunoblot analysis using antibodies against GFP (Fig 1A, left and middle panels), except recombinant EV-A71 2A protein. To resolve this problem, recombinant EV-A71 2A protein was expressed in a bacterial expression system and assessed by immunoblotting with Coomassie brilliant blue R-250 (Fig 1A, middle panel).

EV-A71 virions were purified from the supernatants of cells infected with EV-A71 from a fatal case in Peninsula Malaysia (strain UH1/PM/1997) [3]. EV-A71 virion lysates were prepared under reducing conditions and were then assessed by immunoblot analysis with EV-A71-specific mAb 3323 and mAb 979 (Fig 1A, right panel). Both EV-A71-specific mAb detected VP0 and VP2 proteins, and mAb 979 further detected P1 proteins, VP4+VP2+VP3 proteins and an unidentified protein at approximately 25kDa that could be the proteolytic product of P1 proteins.

Using cell lysates from different EV-A71 proteins, we found that EV-A71-specific IgM antibody from both groups of serum (acute infection with no neutralization, Fig 1B, and acute infection with high neutralization, Fig 1C) recognized all structural proteins (VP1 to VP4) and non-structural proteins 2A, 3C and 3D. EV-A71-specific IgG antibody from acute infection with high neutralization and convalescent sera recognized all structural and non-structural proteins (Fig 1D and 1E). As a parallel control, all these patient sera recognized VP0 protein and an unidentified protein at ≈25kDa in the EV-A71 virion proteins.

### Isotyping of EV-A71-specific antibodies

In order to characterize anti-EV-A71 antibody responses during infection, EV-A71-infected patient sera were tested individually with purified EV-A71 virion-based ELISA, and only those with high absorbance values were selected for subsequent analysis. The total IgM and IgG present in EV-A71-infected patient sera were quantified by the purified EV-A71 virion-based ELISA. Acute infection with high neutralization sera had good antibody responses for EV-A71-specific IgM antibody, even at high dilutions (S2A Fig). All human sera showed weak IgG antibody responses at higher dilutions (S2B Fig). IgG1 subclass was the predominant isotype in the EV-A71-infected patient sera and adult sera, and IgG2 subclass response was increased through convalescence (S2C Fig). A stronger IgG3 subclass response was observed in EV-A71-infected children (acute infection with high neutralization and convalescent samples) than in adults. All IgG subclasseswere found at low level in non-HFMD children (negative control) sera.

### Mapping of EV-A71 specific peptides recognized by patients’ antibodies

**Fig 2:**
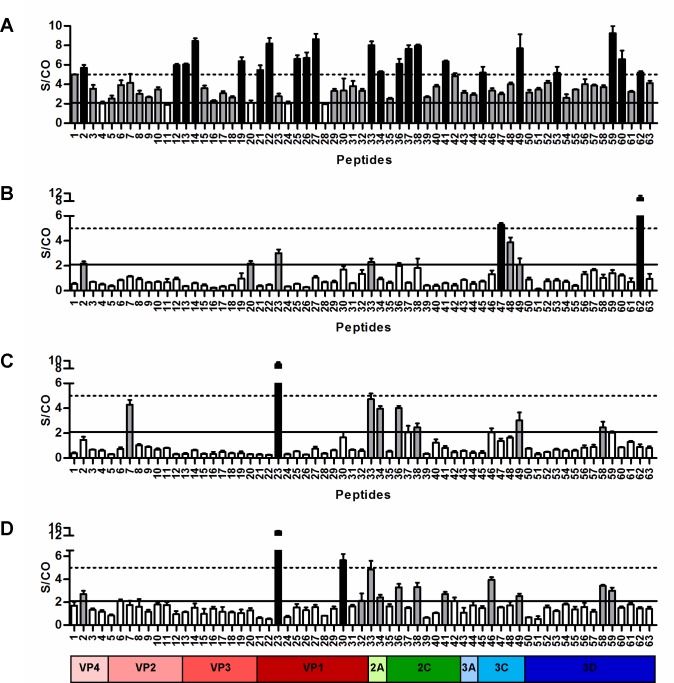
Mapping of EV-A71 B-cell epitopes. Pooled human sera, at optimized dilutions of 1:2000 (IgM) and 1:500 (IgG), were subjected to peptide-based ELISA. (A) Acute infection with high neutralization sera (n = 5) were used for EV-A71-reactive IgM antibody detection. (B) Acute infection with high neutralization sera (n = 5), (C) convalescent sera (n = 3), and (D) adult sera (n = 5) were used for EV-A71-reactive IgG antibody detection. Non-HFMD children sera (n = 4) were used as negative controls. Data are presented as mean ± SD of 3 replicates. Values above the solid black line (S/CO≥2.1) were scored as weakly positive and values above the dotted line (S/CO≥5) were scored as strongly positive reactions. Grey bars represent weakly positive human anti-EV-A71 epitopes and black bars represent strongly positive human anti-EV-A71 epitopes.

A total of 63 biotinylated linear peptides were synthesized as potential EV-A71 B-cell epitopes, based on prediction using EMINI surface accessibility scale or previous publications (S2 Table). Peptide-based ELISAs using these 63 biotinylated peptides were initially performed with 1:2000 dilution and 1:500 dilution pooled sera for IgM and IgG antibodies detection, respectively. Interestingly, a majority of the peptides were recognized as EV-A71-reactive IgM epitopes at S/CO above 2.1 (Fig 2A). Acute infection with high neutralization sera recognized eight EV-A71-reactive IgG epitopes covering VP1, VP3, VP4, 2A, 3C and 3D proteins, with PEP47 and PEP62 as the dominant IgG epitopes (Fig 2B). Convalescent sera (Fig 2C) showed similar EV-A71-reactive IgG epitope profiles as the adult sera (Fig 2D), with eight and thirteen epitopes recognized, respectively. Both sets of sera strongly recognized PEP23 as the dominant epitope, and adult sera also recognized PEP30 as an additional dominant epitope. Both PEP23 and PEP30 are located at the VP1 protein. All identified B-cell linear epitopes are summarized in S2 Table.

**Fig 3:**
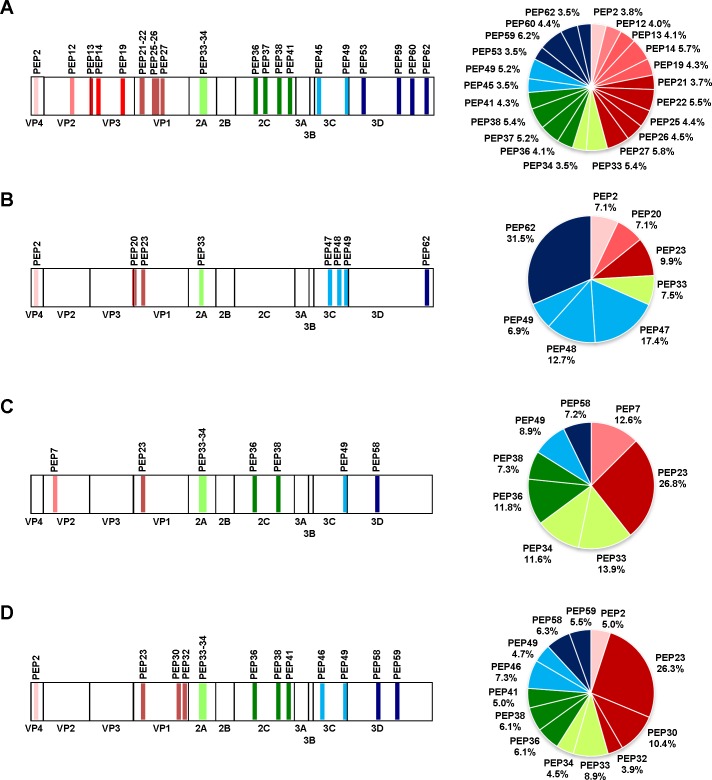
Analysis of anti-EV-A71 antibodies recognizing linear B-cell epitopes. (A) IgM antibody determinants identified from acute infection with high neutralization sera. IgG antibody determinants identified from (B) acute infection with high neutralization sera, (C) convalescent sera, and (D) adult sera. Regions of amino acid sequences corresponding to the identified B-cell epitopes are indicated in the schematic diagrams of the EV-A71 genome. The percentage of antibody recognition contributed by each individual EV-A71 epitope is indicated in the pie charts, and was calculated according to the following equation: % antibody recognition = 100 x (OD values from individual peptide group/sum of the OD values from all peptide groups). In this calculation, the avidity and affinity of the peptides to the sera were assumed to be similar. Peptides are colour-coded according to the respective viral proteins.

For the EV-A71-reactive IgM linear epitopes, only dominant linear epitopes (S/CO ≥5) were selected for further analysis. Results were expressed as the percent contribution of each epitope towards total antibody recognition to the positive peptides used in this study. A total of 22 EV-A71-reactive IgM dominant epitopes were identified and the average percentage of peptide recognition by the sera was very similar (range 3.5% to 6.2%) (Fig 3A). Eight EV-A71-reactive IgG epitopes were recognized by acute infection with high neutralization sera, the most strongly recognized being the PEP62 on the 3D protein and PEP47 on the 3C protein, at 31.5% and 17.4% of total antibody recognition, respectively (Fig 3B). Convalescent sera and adult sera displayed similar antibody recognition profiles. Both had the strongest recognition signals in the VP1 protein, with PEP23 identified as the dominant epitope, with 26.8% (convalescent sera) and 26.3% (adult sera) of the total antibody recognition, respectively (Fig 3C and 3D). Adult sera also recognized PEP30 (located in the VP1 protein) as another dominant epitope, with 10.4% of antibody recognition. No recognizable linear epitopes were found in the VP3 protein for both the pooled sera. Overall, PEP23, PEP33 and PEP49, located within the VP1, 2A and 3C proteins, respectively, were recognized by EV-A71-reactive IgG antibodies from all 3 groups of sera tested. Of these commonly recognized epitopes, PEP23 was identified as the dominant linear epitope, at 9.9, 26.8 and 26.3% of antibody recognition by pooled sera from acute infection with high neutralization, convalescent and adults, respectively; followed by PEP33 (7.5, 13.9 and 8.9%) and PEP49 (6.9, 8.9 and 4.7%).

### Seroprevalence of IgM and IgG in HFMD-infected patients

**Fig 4:**
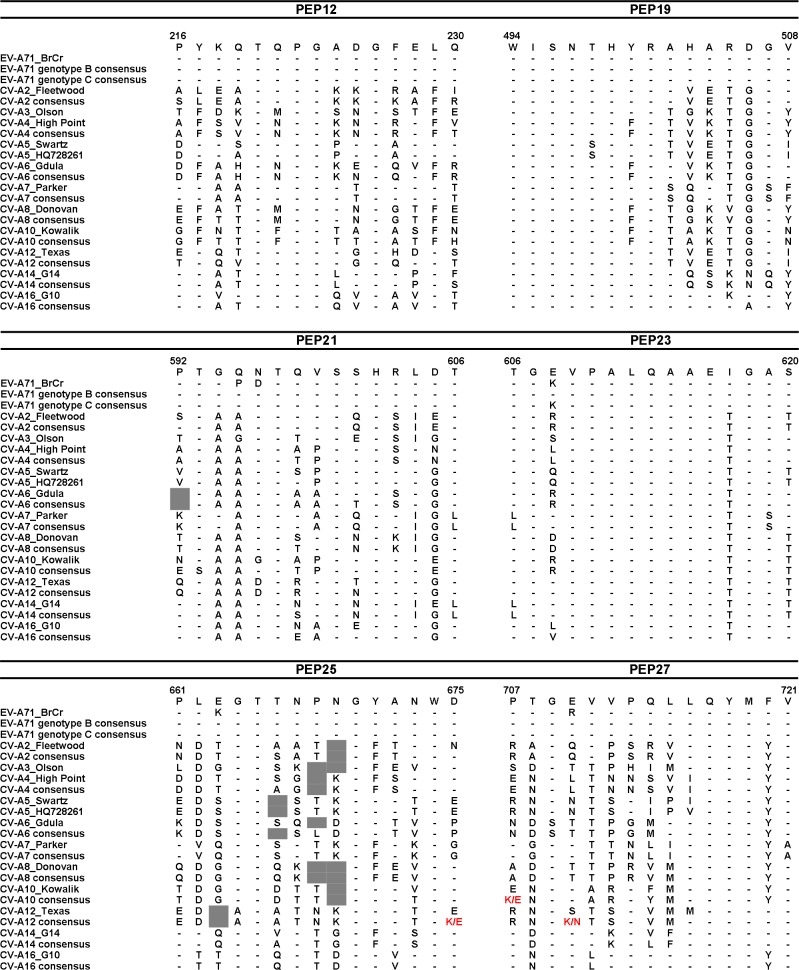
Amino acid sequence alignment of peptides with enteroviruses. The selected peptides (PEP12, PEP19, PEP21, PEP23, PEP25 and PEP27) were aligned to the corresponding sequences from 12 enterovirus prototype and consensus contemporary sequences (EV-A71, CV-A2 to CV-A8, CV-A10, CV-A12, CV-A14 and CV-A16). Conserved amino acids are indicated by a dash (–) and alignment gaps are shown in grey. The consensus sequences represent the current circulating strains while BrCr, Fleetwood, Olson, High Point, Swartz, Gdula, Parker, Donovan, Kowalik, Texas, G14 and G10 are the prototype virus strains.

Of the 22 EV-A71-reactive IgM dominant linear epitopes, 13 peptides were IgM-specific epitopes. These peptide sequences were subsequently aligned to the corresponding sequences from 12 different enteroviruses (EV-A71, CV-A2 to CV-A8, CV-A10, CV-A12, CV-A14 and CV-A16). Epitopes with conserved sequences were removed, and the remaining five epitopes (PEP12, PEP19, PEP21, PEP25 and PEP27), for which EV-A71 sequences varied from other enteroviruses (Fig 4), were selected for study of seroprevalence in HFMD-infected patients.

**Fig 5:**
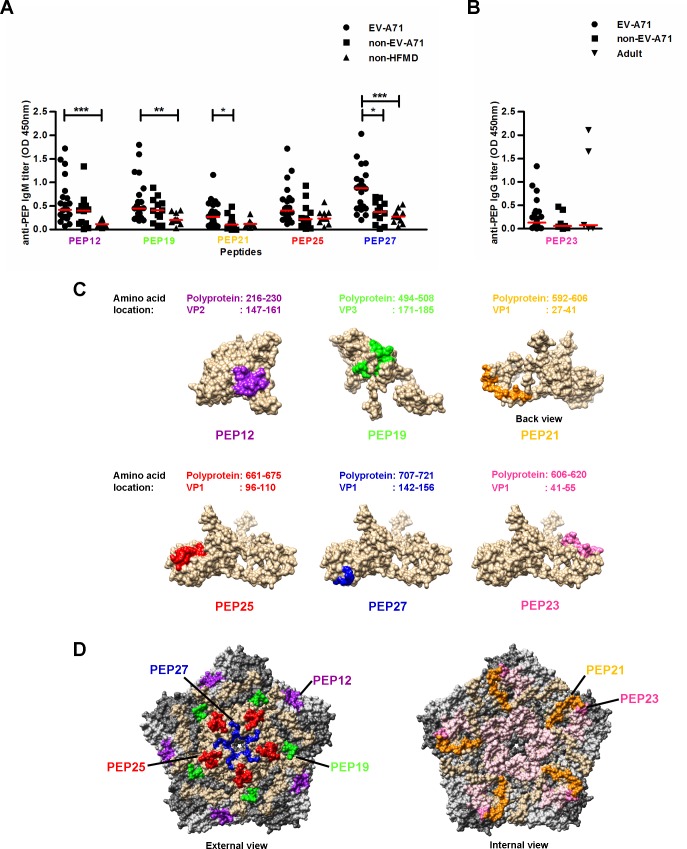
EV-A71-specific IgM and IgG antibody determinants. (A) EV-A71-specific IgM antibody detection in sera (n = 44) at a dilution of 1:2000 was determined by peptide-based ELISA. Sera were categorized into EV-A71-infected patients (n = 22), non-EV-A71 enterovirus-infected patients (n = 12) and non-HFMD patients (n = 10). Red solid lines represent medians. (B) EV-A71-specific IgG antibody detection in sera (n = 38) at a dilution of 1:500 was determined by peptide-based ELISA. Sera were categorized into EV-A71-infected patients (n = 25), non-EV-A71 enterovirus-infected patients (n = 8) and healthy adults (n = 5). Red solid lines represent medians. One-way ANOVA with Kruskal-Wallis test was used for statistical analysis (*P<0.05, **P<0.01, ***P<0.001). (C) Schematic representation of locations of IgM and IgG antibody determinants in VP1, VP2 and VP3 proteins, based on structural data retrieved from PDB records (identifier 3VBS). (D) The EV-A71 pentamer structure (identifier 3VBS) was generated using UCSF Chimera software. The capsid proteins of EV-A71 are shown in brown (VP1), light grey (VP2), dark grey (VP3) and light pink (VP4). The IgM and IgG antibody determinants are indicated in purple (PEP12), green (PEP19), orange (PEP21), red (PEP25), blue (PEP27) and pink (PEP23).

To further validate the specificity and versatility of the selected IgM epitopes as suitable early detection targets, serum samples from patients infected with EV-A71 (n = 22) and non-HFMD children (n = 10) were screened. All IgM linear epitopes showed good responses for EV-A71-infected patient sera, particularly for PEP27, which had the highest mean OD values of 0.85±0.47 (Fig 5A). The OD values of EV-A71-infected patient sera were significantly higher than non-HFMD children sera for PEP12, PEP19, and PEP27, suggesting that these IgM epitopes are suitable for distinguishing EV-A71 and non-HFMD patients. To determine if the EV-A71 IgM epitopes cross-react with antibodies from patients infected with other enteroviruses, peptide-based ELISA was further performed using 12 serum samples from patients infected with non-EV-A71 enteroviruses. Serum samples from patients infected with non-EV-A71 enteroviruses, namely CV-A4, CV-A6, CV-A16, echovirus 7 and untyped enteroviruses, showed cross-reactivity to PEP12, PEP19 and PEP25, suggesting that these IgM epitope recognitions are not EV-A71-specific. Significant differences between the OD values of EV-A71-infected and non-EV-A71-infected patient sera were observed in PEP21 and PEP27 only, suggesting that these IgM epitopes are EV-A71 specific. Overall, PEP27 is the best EV-A71-specific IgM epitope among the five IgM epitopes.

For the EV-A71-specific IgG epitopes, PEP23 was selected for individual serum testing, as it is the dominant epitope that was commonly recognized by the three sets of sera. All individual serum samples selected were positive for EV-A71-specific IgG. No statistical differences were observed, as shown in Fig 5B. These results also suggest the EV-A71 IgG epitope PEP23 may be highly cross-reactive.

Epitope-containing sequences were next mapped onto the available three-dimensional crystal structures of the VP1, VP2 and VP3 proteins (PDB identifier: 3VBS). For the EV-A71-specific IgM epitopes, PEP12 is located in the solvent-exposed region of VP2, whereas PEP19 is partially embedded in the VP3 protein (Fig 5C). Similar analyses for the VP1 protein revealed that PEP21 and PEP25 are located in the solvent-exposed region, while PEP27 is partially buried in the VP1 protein. The EV-A71-specific IgG epitope PEP23 is prominently exposed on the surface of the VP1 protein. A pentamer structure of the capsid proteins of EV-A71 was constructed (Fig 5D). PEP19, PEP25 and PEP27 were exposed on the EV-A71 surface, whereas PEP21 and PEP23 were buried within the EV-A71 pentamer.

## Discussion

Understanding the immune responses against EV-A71 is important for the development of diagnostic tools and potential subunit vaccine candidates. In the present study, the first comprehensive analysis of antibody responses against structural and non-structural proteins of EV-A71 was performed. Using EV-A71-infected patient serum samples from HFMD outbreaks in Malaysia [28], all viral structural proteins (VP1-VP4) were found to be targets for EV-A71-specific IgM and IgG antibodies. EV-A71-specific IgM antibody also recognized viral non-structural proteins 2A, 3C and 3D, whereas EV-A71-specific IgG antibody recognized all the non-structural proteins. For the foot-and-mouth disease virus, another picornavirus, non-structural proteins are used as antigens in diagnostic ELISA to distinguish infected animals from vaccinated animals due to their high immunogenicity [33]. Infected animals produce antibodies against both structural and non-structural proteins, whereas vaccinated animals only develop antibodies against structural proteins since the inactivated vaccine has no replicating virus [34]. Among all the non-structural proteins, 3ABC was shown to be the most appropriate antigen to distinguish infection from vaccination [35–37]. Three EV-A71 vaccines have completed phase III clinical trials and may be available soon [38–40], and there will be a need for assays to serologically differentiate vaccinated from naturally-infected individuals. Our study suggests that potential antigens for this purpose are non-structural proteins 2A, 3C and 3D, which are recognized by naturally-acquired IgM; and 2A, 2B, 2C, 3AB, 3C and 3D, which are recognized by naturally-acquired IgG from EV-A71-infected patient sera. Further investigation will be required to determine which protein(s) should be used as antigens for diagnostic assays.

The different roles of human IgG subclasses in neutralization and antibody-dependent enhancement are well documented in dengue and West Nile virus studies [41,42]. In EV-A71, IgG1 subclass demonstrated the strongest neutralizing ability and is found in human intravenous immunoglobulin [43]. In the present study, IgG1 subclass was identified as the predominant isotype in EV-A71-infected patient sera. It has been shown that IgG3 subclass did not have neutralizing activity but enhanced EV-A71 infection *in vitro* [44]. Interestingly, a stronger IgG3 subclass response was observed in EV-A71-infected children than in adults, and this could imply poorer neutralizing antibody protection in children compared to adults.

IgM detection is important for early diagnosis of infectious diseases. A total of 63 biotinylated peptides were synthesized to determine EV-A71-reactive IgM linear epitopes using peptide-based ELISAs. Linear epitopes instead of discontinuous epitopes were the focus since they are more easily identifiable in a medium-throughput approach [45]. Interestingly, 92.1% (58/63) of the peptides were recognized by IgM antibody from EV-A71-infected patient sera. Overall, 22 dominant human anti-EV-A71 IgM linear epitopes were identified, suggesting that anti-EV-A71 IgM responses against multiple epitopes are induced during EV-A71 infection [24]. The dominant linear epitopes with S/CO levels of ≥5 were PEP12, PEP14 and PEP22, which were mapped to similar locations as the human IgM epitopes VP2-50, VP3-10, VP3-12 and VP1-14 reported in a previous study [24]. To determine the seroprevalence of IgM against these EV-A71 epitopes, five unique epitopes with least similarity to other enteroviruses were screened with individual serum testing by using peptide-based ELISAs. Non-HFMD children sera were used to eliminate the non-specific epitopes, and these samples are likely from patients without active EV-A71 infection based on the negative results obtained from EV-A71 IgM-capture ELISA and neutralization titers of <8. The OD values of EV-A71-infected patient sera tested against PEP12, PEP19 and PEP27 were significantly higher than the OD values of non-HFMD children sera, suggested that these IgM epitopes are enterovirus-reactive epitopes. Cross-reactivity was observed in PEP12 and PEP19, suggesting that these epitope recognitions are not EV-A71-specific. The high cross-reactivity is likely due to previous exposure to other enteroviruses, which cannot be ruled out. However, since children can be infected with HFMD multiple times, it also suggests that these cross-reactive epitopes will not result in cross-protection. Significant differences between the OD values of EV-A71-infected and non-EV-A71 enterovirus-infected patient sera were observed for PEP27, suggesting that this epitope is EV-A71-specific. Overall, PEP27 mapped to VP1 142–156 is the best candidate for an EV-A71-specific IgM epitope. PEP27 was found to be mapped to a similar location as the reported CD4^+^ T-cell epitopes SP2 and VP1-20 [46,47], suggesting that PEP27 is a potential EV-A71 B-cell and T-cell epitope. However, the OD values of PEP27 in non-HFMD children sera was higher than the remaining four IgM epitopes, suggesting that the cut-off could be improved. High population immunity to EV-A71 has been reported in Malaysian children, with EV-A71 seropositive rates in non-HFMD urban children increasing gradually from 47.1% at 1–3 years to 75.0% at 13–17 years [29], suggesting that true non-HFMD sera are required to establish a more reliable cut-off.

Different human IgG epitopes were recognized by patient serum samples from acute infection with high neutralization and convalescence. Using EV-A71-infected patient sera from acute infection with high neutralization, we observed that PEP47 and PEP62, located at non-structural proteins 3C and 3D, respectively, were identified as dominant anti-EV-A71 IgG linear epitopes. Convalescent sera showed similar EV-A71-reactive IgG epitope profiles as adult sera, as both sera strongly recognized PEP23 as the dominant IgG linear epitope, and adult sera also recognized PEP30 as an additional dominant linear epitope. Surprisingly, all the previously reported anti-EV-A71 IgG epitopes identified in rabbit and mouse sera (in our panel of 63 peptides) were not recognized by EV-A71-infected patient sera, except PEP20, which mapped to a similar location as the reported VP1-01 epitope [22]. The synthetic peptide SP70, located at amino acids 208–222 in VP1, has been identified as a neutralizing and protective EV-A71-specific B-cell epitope in animals [16,22,48]. Several studies have demonstrated that neutralizing mAbs generated by EV-A71-immunized mice can recognize SP70 [17–20,22]. In addition, VP2-28 (amino acids 136–150 in VP2) is a cross-neutralization epitope recognized by commercial mAb 979 and mAbs generated by immunized mice [22, 2,49]. In the present study, EV-A71-infected human sera from acute and convalescent stages failed to recognize the EV-A71-specific neutralizing epitopes SP70 and VP2-28, represented by PEP29 and PEP10, respectively. Antisera from rabbits injected with formalin-inactivated EV-A71 only recognized VP2-28 peptide, while mouse antisera only recognized VP1-43 (amino acids 211–225 in VP1; similar positions with SP70) [22]. The findings from our and other studies suggest that human sera may target different immunogenic epitopes compared with sera from immunized mice and rabbit. Despite the epitope-specific responses, all animal antisera showed virus neutralizing activities [22].

Interestingly, PEP23 mapped to VP1 41–55 was recognized by all anti-EV-A71 IgG antibodies from patient and adult sera. This PEP23 was the previously reported human IgG epitope VP1-15 [24]. Although this long-lasting immune response of anti-EV-A71 IgG antibody against PEP23 would make it an attractive candidate for seroepidemiology studies, cross-reactivity was observed against PEP23, suggesting that this epitope is not EV-A71 specific. Further improvements such as the use of longer peptides or multiple peptides may reduce the cross-reactivity in PEP23. Further development of a good peptide ELISA will be valuable for the evaluation of EV-A71 vaccine immunogenicity. In future, it would also be interesting to determine the presence of conformational epitopes within the structural and non-structural proteins.

In summary, PEP27 mapped at VP1 142–156 was identified as an EV-A71 IgM-specific immunodominant epitope, and is a candidate for use in a diagnostic IgM assay that differentiates EV-A71 from other causes of HFMD. This would be a valuable tool for early public health responses. PEP23 mapped to VP1 41–55 was identified as an IgG cross-reactive immunodominant epitope. Overall, the structural protein VP1 is the main immunodominant site targeted by anti-EV-A71 IgM and IgG antibodies. In addition, epitopes against non-structural proteins were also detected. This study has provided useful insights into human immune responses to different EV-A71 epitopes at different stages of infection.

## Supporting Information

S1 Fig Schematic illustration of 63 synthetic biotinylated peptides. The distribution of the peptides is shown in the (A) EV-A71 whole genome, and (B) in each EV-A71 gene.
(TIF)

S2 Fig Measurement of EV-A71-specific antibodies. (A) EV-A71-specific IgM antibody titers and (B) EV-A71-specific IgG antibody titers were determined by virion-based ELISAs. EV-A71-infected patient pooled sera were assayed by serial dilution and subjected to virion-based ELISA. (C) EV-A71-specific IgG isotype titers in pooled sera were determined at dilutions of 1:100 using specific secondary antibodies. Non-HFMD children sera were used as negative controls. Data are presented as mean ± SD of 3 replicates.
(TIF)

S1 Table Detailed information of human serum grouping. (DOCX)

S2 Table List of the 63 synthetic biotinylated peptides and their reactions with serum samples. (DOCX)
