# Supplementary figures and images for: Immunodominant IgM and IgG Epitopes Recognized by Antibodies Induced in Enterovirus A71-Associated Hand, Foot and Mouth Disease Patients
Source: PLoS One. 2016 Nov 2;11(11):e0165659. doi: 10.1371/journal.pone.0165659 (PMC5091889; doi:10.1371/journal.pone.0165659)

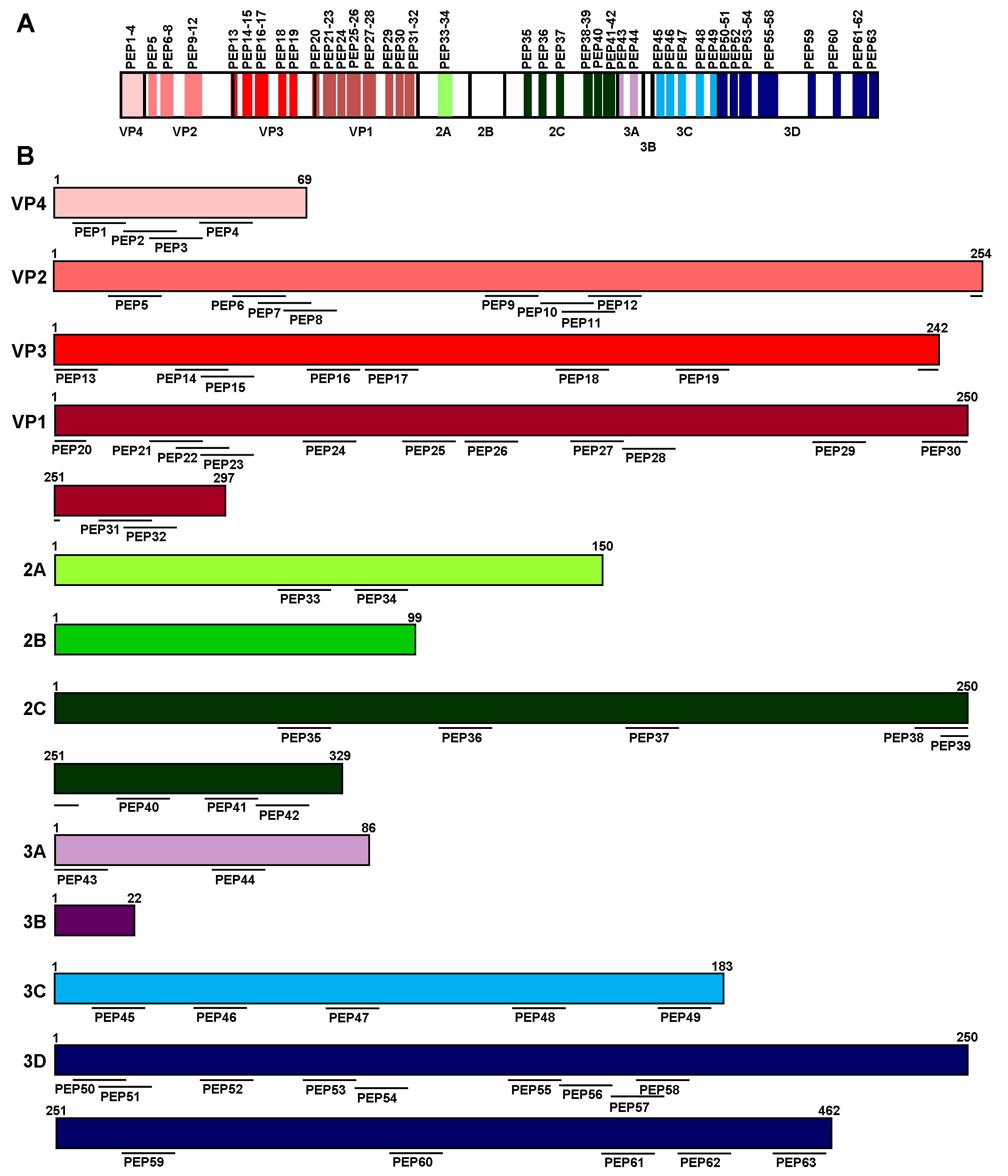

Supplement: S1 Fig — The distribution of the peptides is shown in the (A) EV-A71 whole genome, and (B) in each EV-A71 gene. (TIF) [file pone.0165659.s001.tif]

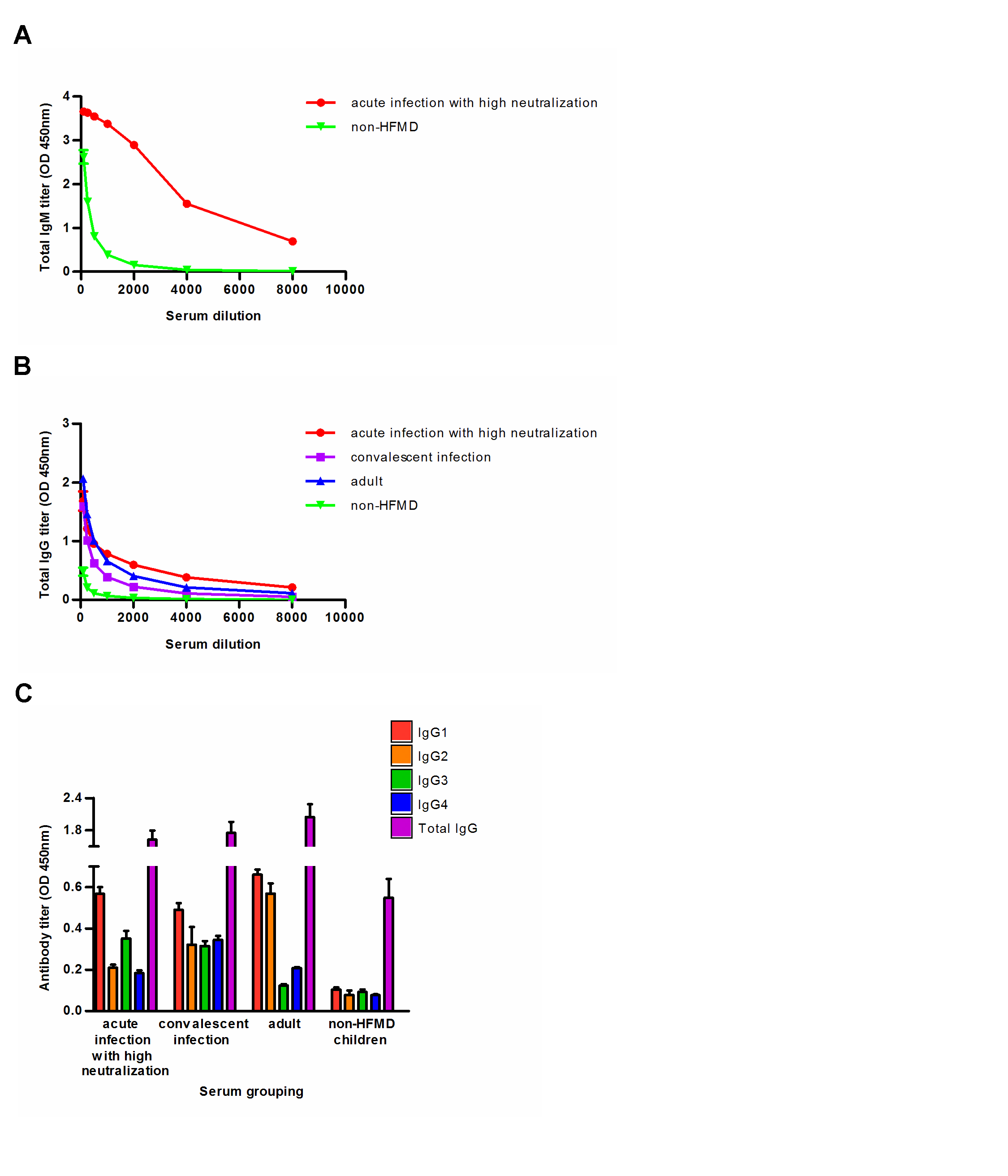

Supplement: S2 Fig — (A) EV-A71-specific IgM antibody titers and (B) EV-A71-specific IgG antibody titers were determined by virion-based ELISAs. EV-A71-infected patient pooled sera were assayed by serial dilution and subjected to virion-based ELISA. (C) EV-A71-specific IgG isotype titers in pooled sera were determined at dilutions of 1:100 using specific secondary antibodies. Non-HFMD children sera were used as negative controls. Data are presented as mean ± SD of 3 replicates. (TIF) [file pone.0165659.s002.tif]
