# Supplementary material for: Immunodominant IgM and IgG Epitopes Recognized by Antibodies Induced in Enterovirus A71-Associated Hand, Foot and Mouth Disease Patients
Source: PLoS One. 2016 Nov 2;11(11):e0165659. doi: 10.1371/journal.pone.0165659 (PMC5091889; doi:10.1371/journal.pone.0165659)
Supplement: S1 Table — (DOCX) [file pone.0165659.s003.docx]

**S1 Table: Detailed information of human serum grouping**

|  | **Number of samples** | **Age (years)** | **Presence of** | | **Neutralization titer** | **OD range** | |
| --- | --- | --- | --- | --- | --- | --- | --- |
|  |  |  | **IgM** | **IgG** |  | **IgM ^a^** | **IgG ^b^** |
| **(A) Overall** |  |  |  |  |  |  |  |
| HFMD patient | 43 | 0.17 - 10 | - / + | - / + | <1:8 / ≥1:8 | 0.00 - 2.20 | 0.17 - 1.76 |
| Non-HFMD (children) | 10 | 0.67 - 11 | - | - / + | <1:8 / ≥1:8 | 0.00 - 0.05 | 0.22 - 1.77 |
| Non-HFMD (adult) | 5 | 25 - 39 | - | + | ≥1:32 | 0.04 - 0.11 | 0.52 - 2.47 |
|  |  |  |  |  |  |  |  |
| **(B) Western blot: Antigen recognition** | |  |  |  |  |  |  |
| Acute infection with no neutralization | 2 | 1.5 - 2 | + | + | <1:8 | 0.31 - 1.03 | 0.54 - 0.79 |
| Acute infection with high neutralization | 12 | 1 - 4 | + | + | ≥1:32 | 0.42 - 2.20 | 0.63 - 1.32 |
| Convalescent | 5 | 1 - 4.25 | - | + | ≥1:8 | 0.02 - 0.10 | 0.75 - 1.76 |
|  |  |  |  |  |  |  |  |
| **(C) ELISA: Epitope mapping** | |  |  |  |  |  |  |
| Acute infection with high neutralization | 5 | 1.83 - 4 | + | + | ≥1:32 | 0.76 - 1.62 | 0.98 - 1.32 |
| Convalescent | 3 | 1 - 4.25 | - | + | ≥1:8 | 0.02 - 0.03 | 1.07 - 1.76 |
| Non-HFMD (children) | 4 | 0.67 - 4 | - | - / + | <1:8 / ≥1:8 | 0.00 - 0.01 | 0.22 - 1.50 |
| Non-HFMD (adult) | 5 | 25 - 39 | - | + | ≥1:32 | 0.04 - 0.11 | 0.52 - 2.47 |
|  |  |  |  |  |  |  |  |
| **(D) ELISA: IgM seroprevalence** | |  |  |  |  |  |  |
| EV-A71 | 22 | 1 - 4.67 | + | - / + | <1:8 / ≥1:8 | 0.28 - 2.20 | 0.33 - 1.33 |
| Non-EV-A71 enteroviruses | 12 | 0.17 - 10 | - | - / + | <1:8 / ≥1:8 | 0.00 - 0.11 | 0.17 - 1.70 |
| Non-HFMD (children) | 10 | 0.67 - 11 | - | - / + | <1:8 / ≥1:8 | 0.00 - 0.05 | 0.22 - 1.77 |
|  |  |  |  |  |  |  |  |
| **(E) ELISA: IgG seroprevalence** | |  |  |  |  |  |  |
| EV-A71 | 25 | 1 - 7 | - / + | + | <1:8 / ≥1:8 | 0.02 - 2.20 | 0.46 - 1.76 |
| Non-EV-A71 enteroviruses | 7 | 0.67 - 9.33 | - / + | + | <1:8 / ≥1:8 | 0.02 - 1.74 | 0.40 - 1.70 |
| Non-HFMD (adult) | 5 | 25 - 39 | - | + | ≥1:32 | 0.04 - 0.11 | 0.52 - 2.47 |
|  |  |  |  |  |  |  |  |

^a^ IgM was detected using commercial EV-A71 IgM-capture ELISA

b IgG was detected using virion-based ELISA
