# Supplementary material for: Immunodominant IgM and IgG Epitopes Recognized by Antibodies Induced in Enterovirus A71-Associated Hand, Foot and Mouth Disease Patients
Source: PLoS One. 2016 Nov 2;11(11):e0165659. doi: 10.1371/journal.pone.0165659 (PMC5091889; doi:10.1371/journal.pone.0165659)
Supplement: S2 Table — (DOCX) [file pone.0165659.s004.docx]

**S2 Table: List of the 63 synthetic biotinylated peptides and their reactivity with human serum**

| **Name** | **Sequence** | **Region** | **Amino acid position** | | **IgM epitope**  **(acute^a^)** | **IgG epitope**  **(acute^a^)** | **IgG epitope (convalescent)** | **IgG epitope**  **(adult)** | **Prediction / reference** |
| --- | --- | --- | --- | --- | --- | --- | --- | --- | --- |
|  |  |  | **Polyprotein** | **Specific protein** |  |  |  |  |  |
| PEP1 | STQRSGSHENSNSAT | VP4 | 6-20 | 6-20 | + | - | - | - | Predicted by EMINI [31] |
| PEP2 | TEGSTINYTTINYYK | VP4 | 20-34 | 20-34 | ++ | + | - | + | Predicted by EMINI [31] |
| PEP3 | YTTINYYKDSYAATA | VP4 | 27-41 | 27-41 | + | - | - | - | Predicted by EMINI [31] |
| PEP4 | AGKQSLKQDPDKFAN | VP4 | 41-55 | 41-55 | - | - | - | - | Predicted by EMINI [31] |
| PEP5 | LTIGNSTITTQEAAN | VP2 | 85-99 | 16-30 | + | - | - | - | Predicted by EMINI [31] |
|  |  |  |  |  |  |  |  |  | VP2-6 [24] |
| PEP6 | VDKPTRPDVSVNRFY | VP2 | 119-133 | 50-64 | + | - | - | - | Predicted by EMINI [31] |
| PEP7 | DVSVNRFYTLDTKLW | VP2 | 126-140 | 57-71 | + | - | + | - | VP2-21 [24] |
| PEP8 | YTLDTKLWEKSSKGW | VP2 | 133-147 | 64-78 | + | - | - | - | Predicted by EMINI [31] |
| PEP9 | QGALLVAILPEYVIG | VP2 | 188-202 | 119-133 | + | - | - | - | VP2-40 [24] |
| PEP10 | TVAGGTGTEDSHPPY | VP2 | 203-217 | 134-148 | + | - | - | - | Predicted by EMINI [31] |
|  |  |  |  |  |  |  |  |  | VP2-27 [22] |
|  |  |  |  |  |  |  |  |  | VP2-28 [22] |
| PEP11 | GTEDSHPPYKQTQPG | VP2 | 209-223 | 140-154 | - | - | - | - | Predicted by EMINI [31] |
|  |  |  |  |  |  |  |  |  | VP2-29 [22] |
| PEP12 | PYKQTQPGADGFELQ | VP2 | 216-230 | 147-161 | ++ | - | - | - | Predicted by EMINI [31] |
|  |  |  |  |  |  |  |  |  | VP2-50 [24] |
| PEP13 | VTQGFPTEPKPGTNQ | VP2-VP3 | 321-335 | 252-254 (VP2),  1-12 (VP3) | ++ | - | - | - | Predicted by EMINI [31] |
| PEP14 | IHIPGEVRNLLELCQ | VP3 | 357-371 | 34-48 | ++ | - | - | - | VP3-10 [24] |
|  |  |  |  |  |  |  |  |  | VP3-12 [24] |
| PEP15 | RNLLELCQVETILEV | VP3 | 364-378 | 41-55 | + | - | - | - | VP3-15 [24] |
| PEP16 | RFPVSAQAGKGELCA | VP3 | 393-407 | 70-84 | + | - | - | - | VP3-24 [24] |
| PEP17 | FRADPGRDGPWQSTM | VP3 | 409-423 | 86-100 | + | - | - | - | Predicted by EMINI [31] |
| **Name** | **Sequence** | **Region** | **Amino acid position** | | **IgM epitope**  **(acute^a^)** | **IgG epitope**  **(acute^a^)** | **IgG epitope (convalescent)** | **IgG epitope**  **(adult)** | **Prediction / reference** |
|  |  |  | **Polyprotein** | **Individual protein** |  |  |  |  |  |
| PEP18 | PGGPLPKDRATAMLG | VP3 | 461-475 | 138-152 | + | - | - | - | Predicted by EMINI [31] |
| PEP19 | WISNTHYRAHARDGV | VP3 | 494-508 | 171-185 | ++ | - | - | - | Predicted by EMINI [31] |
| PEP20 | QTASIQGDRVADVIE | VP3-VP1 | 560-574 | 237-242 (VP3),  1-9 (VP1) | - | + | - | - | Predicted by EMINI [31] |
|  |  |  |  |  |  |  |  |  | VP1-01 [22] |
| PEP21 | PTGQNTQVSSHRLDT | VP1 | 592-606 | 27-41 | ++ | - | - | - | Predicted by EMINI [31] |
| PEP22 | VSSHRLDTGEVPALQ | VP1 | 599-613 | 34-48 | ++ | - | - | - | VP1-14 [24] |
| PEP23 | TGEVPALQAAEIGAS | VP1 | 606-620 | 41-55 | + | + | ++ | ++ | VP1-15 [24] |
| PEP24 | VLNSHSTAETTLDSF | VP1 | 634-648 | 69-83 | - | - | - | - | Predicted by EMINI [31] |
| PEP25 | PLEGTTNPNGYANWD | VP1 | 661-675 | 96-110 | ++ | - | - | - | Predicted by EMINI [31] |
|  |  |  |  |  |  |  |  |  | BC loop |
|  |  |  |  |  |  |  |  |  | VP1-31 [24] |
| PEP26 | ITGYAQMRRKVELFT | VP1 | 678-692 | 113-127 | ++ | - | - | - | Predicted by EMINI [31] |
| PEP27 | PTGEVVPQLLQYMFV | VP1 | 707-721 | 142-156 | ++ | - | - | - | Predicted by EMINI [31] |
|  |  |  |  |  |  |  |  |  | DE loop |
| PEP28 | VPPGAPKPESRESLA | VP1 | 721-735 | 156-170 | - | - | - | - | Predicted by EMINI [31] |
|  |  |  |  |  |  |  |  |  | SP55 [16] |
|  |  |  |  |  |  |  |  |  | VP1-54 [24] |
| PEP29 | YPTFGEHKQEKDLEY | VP1 | 773-787 | 208-222 | + | - | - | - | Predicted by EMINI [31] |
|  |  |  |  |  |  |  |  |  | SP70 [16] |
|  |  |  |  |  |  |  |  |  | VP1-71 [24] |
|  |  |  |  |  |  |  |  |  | VP1-42 [22] |
|  |  |  |  |  |  |  |  |  | VP1-43 [22] |
| PEP30 | VGSSKSKYPLVVRIY | VP1 | 803-817 | 238-252 | + | - | - | ++ | HI loop |
| PEP31 | PRPMRNQNYLFKANP | VP1 | 828-842 | 263-277 | + | - | - | - | Predicted by EMINI [31] |
| PEP32 | NYLFKANPNYAGNSI | VP1 | 835-849 | 270-284 | + | - | - | + | Predicted by EMINI [31] |
| **Name** | **Sequence** | **Region** | **Amino acid position** | | **IgM epitope**  **(acute^a^)** | **IgG epitope**  **(acute^a^)** | **IgG epitope (convalescent)** | **IgG epitope**  **(adult)** | **Prediction / reference** |
|  |  |  | **Polyprotein** | **Individual protein** |  |  |  |  |  |
| PEP33 | VYYCNSKRKHYPVSF | 2A | 924-938 | 62-76 | ++ | + | + | + | Predicted by EMINI [31] |
| PEP34 | YVEASEYYPARYQSH | 2A | 945-959 | 83-97 | ++ | - | + | + | Predicted by EMINI [31] |
| PEP35 | NLEQSAASQEDLEAM | 2C | 1173-1187 | 62-76 | + | - | - | - | Predicted by EMINI [31] |
| PEP36 | KRMNNYMQFKSKHRI | 2C | 1217-1231 | 106-120 | ++ | - | + | + | Predicted by EMINI [31] |
| PEP37 | LPPDPDHFDGYKQQV | 2C | 1268-1282 | 157-171 | ++ | - | - | - | Predicted by EMINI [31] |
| PEP38 | DAIRRRFYMDCDIEV | 2C | 1347-1361 | 236-250 | ++ | - | + | + | Predicted by EMINI [31] |
| PEP39 | YMDCDIEVTDSYKTD | 2C | 1354-1368 | 243-257 | + | - | - | - | Predicted by EMINI [31] |
| PEP40 | KLCSENNTANFKRCS | 2C | 1379-1393 | 268-282 | + | - | - | - | Predicted by EMINI [31] |
| PEP41 | LRDRKSKVRYSVDTV | 2C | 1403-1417 | 292-306 | ++ | - | - | + | Predicted by EMINI [31] |
| PEP42 | VVSELIREYNSRSAI | 2C | 1417-1431 | 306-320 | + | - | - | - | Predicted by EMINI [31] |
| PEP43 | GPPKFRPIRISLEEK | 3A | 1441-1455 | 1-15 | + | - | - | - | Predicted by EMINI [31] |
| PEP44 | IPETPTNVERHLNRA | 3A | 1484-1498 | 44-58 | + | - | - | - | Predicted by EMINI [31] |
| PEP45 | LRRNIRQVQTDQGHF | 3C | 1559-1573 | 11-25 | ++ | - | - | - | Predicted by EMINI [31] |
| PEP46 | RHSQPGKTIWVEHKL | 3C | 1587-1601 | 39-53 | + | - | - | + | Predicted by EMINI [31] |
| PEP47 | VTLDTNEKFRDITKF | 3C | 1623-1637 | 75-89 | + | ++ | - | - | Predicted by EMINI [31] |
| PEP48 | NLSGKPTHRTMMYNF | 3C | 1674-1688 | 126-140 | + | + | - | - | Predicted by EMINI [31] |
| PEP49 | GRQGFCAGLKRSYFA | 3C | 1714-1728 | 166-180 | ++ | + | + | + | Predicted by EMINI [31] |
| PEP50 | VKPNKETGRLNINGP | 3D | 1737-1751 | 6-20 | + | - | - | - | Predicted by EMINI [31] |
| PEP51 | GRLNINGPTRTKLEP | 3D | 1744-1758 | 13-27 | + | - | - | - | Predicted by EMINI [31] |
| PEP52 | AVLTSKDPRLEVDFE | 3D | 1772-1786 | 41-55 | + | - | - | - | Predicted by EMINI [31] |
| PEP53 | EPDEYVTQAALHYAN | 3D | 1800-1814 | 69-83 | ++ | - | - | - | Predicted by EMINI [31] |
| PEP54 | NQLKQLDINTSKMSM | 3D | 1814-1828 | 83-97 | + | - | - | - | Predicted by EMINI [31] |
| PEP55 | IKKRDILDPTTRDVS | 3D | 1856-1870 | 125-139 | + | - | - | - | Predicted by EMINI [31] |
| PEP56 | SKMKFYMDKYGLDLP | 3D | 1870-1884 | 139-153 | + | - | - | - | Predicted by EMINI [31] |
| PEP57 | PYSTYVKDELRSLDK | 3D | 1884-1898 | 153-167 | + | - | - | - | Predicted by EMINI [31] |
| **Name** | **Sequence** | **Region** | **Amino acid position** | | **IgM epitope**  **(acute^a^)** | **IgG epitope**  **(acute^a^)** | **IgG epitope (convalescent)** | **IgG epitope**  **(adult)** | **Prediction / reference** |
|  |  |  | **Polyprotein** | **Individual protein** |  |  |  |  |  |
| PEP58 | DELRSLDKIKKGKSR | 3D | 1891-1905 | 160-174 | + | - | + | + | Predicted by EMINI [31] |
| PEP59 | INHTHHVYRNKTYCV | 3D | 2000-2014 | 269-283 | ++ | - | - | + | Predicted by EMINI [31] |
| PEP60 | LELAKTGKEYGLTMT | 3D | 2073-2087 | 342-356 | ++ | - | - | - | Predicted by EMINI [31] |
| PEP61 | ESIRWTKDARNTQDH | 3D | 2131-2145 | 400-414 | + | - | - | - | Predicted by EMINI [31] |
| PEP62 | LAWHNGKDEYEKFVS | 3D | 2152-2166 | 421-435 | ++ | ++ | - | - | Predicted by EMINI [31] |
| PEP63 | AIPNFENLRRNWLEL | 3D | 2178-2192 | 447-461 | + | - | - | - | Predicted by EMINI [31] |

^a^ acute infection with high neutralization

++, strongly positive ELISA reaction with human anti-EV-A71 serum

+, weakly positive ELISA reaction with human anti-EV-A71 serum

-, negative ELISA reaction with human anti-EV-A71 serum
